# Pure intravascular recurrence of CD5-positive diffuse large B-cell lymphoma primarily arising from the nasal cavities

**DOI:** 10.1186/s13000-018-0724-x

**Published:** 2018-07-24

**Authors:** Rieko Kano, Hiroaki Masaie, Akihisa Hino, Hironao Yasuoka, Shigenori Nagata, Jun Ishikawa, Shin-ichi Nakatsuka

**Affiliations:** 1 Department of Diagnostic Pathology and Cytology, Osaka International Cancer Institute Hospital, 3-1-69 Otemae, Chuo-ku, Osaka, 541-8567 Japan; 2 Department of Hematology, Osaka International Cancer Institute Hospital, 3-1-69 Otemae, Chuo-ku, Osaka, 541-8567 Japan; 3 Department of Pathology, Osaka Police Hospital, 10-31 Kitayama-Town, Tenno-ji-ku, Osaka, 543-0035 Japan

**Keywords:** CD5-positive diffuse large B-cell lymphoma, Intravascular large B-cell lymphoma, Asian variant, Random skin biopsy

## Abstract

**Background:**

CD5-positive diffuse large B-cell lymphoma (DLBCL) and intravascular large B-cell lymphoma (IVL) are recognized as rare subsets of large B-cell lymphoma with poor prognosis. These two categories have similar clinicopathological features suggesting that they might overlap.

**Case presentation:**

We present a case of a 72-year-old man with submental tumors. Positron emission tomography/computed tomography (PET/CT) showed tumors in the nasal and paranasal region and multiple submental and jugular swollen lymph nodes with abnormal uptake of ^18^F-fluorodeoxyglucose (FDG). Histology of biopsy from nasal tumors showed diffuse infiltration of large lymphoid cells, which showed positive expressions for CD20, CD79a, CD5 and negative for CD3 on immunohistochemistry. Thus, a CD5-positive DLBCL was diagnosed. After administration of 8 cycles of R-THPCOP (rituximab, pirarubicin, cyclophosphamide, vincristine and prednisolone), complete remission was achieved. Eight months after the first chemotherapy dose, local recurrence occurred. After salvage chemotherapy, nasal and paranasal tumors and lymph node swelling disappeared on PET/CT images, although the patient suffered from respiratory disturbance. A random skin biopsy revealed IVL, which was consistent with intravascular recurrence of CD5-positive DLBCL. Bone marrow smears showed hemophagocytosis.

**Conclusion:**

We present a rare case of primary CD5-positive DLBCL that relapsed as pure IVL after chemotherapy. Our case suggests that CD5-positive DLBCL is closely related to IVL.

## Background

Diffuse large B-cell lymphoma (DLBCL) is the most common subtype of B-cell lymphoma with heterogeneous biological and clinical features. In a small subset of DLBCL cases, CD5, a pan-T-cell surface marker, is expressed. CD5 is also expressed in most cases of chronic lymphocytic leukemia (CLL) and mantle cell lymphoma. CD5-positive DLBCL is divided into de novo and secondary groups [1]. De novo CD5-positive DLBCL is associated with high-risk clinical features, performance status > 1, bone marrow involvement, and central nervous system (CNS) recurrence [2–5]. The majority of CD5-positive DLBCL cases show an activated B-cell subtype phenotype on genetic profiling [2, 6] and non-germinal center B-cell phenotype on immunohistochemistry [4, 7]. De novo CD5-positive DLBCL frequently shows partial intravascular or intrasinusoidal infiltration [5], which is similar to the histological features of intravascular large B-cell lymphoma (IVL). Conversely, IVL often shows CD5 expression on immunohistochemistry, and the frequency of CD5-positive IVL cases is high (22–38%) [8–10]; this is particularly true for the Asian variant of IVL. These findings suggest a close relationship between CD5-positive DLBCL and IVL. Here we report a case of primary nasal CD5-positive DLBCL that relapsed as pure IVL with hemophagocytic lymphohistiocytosis after rituximab-based chemotherapy.

## Case presentation

**Fig. 1:**
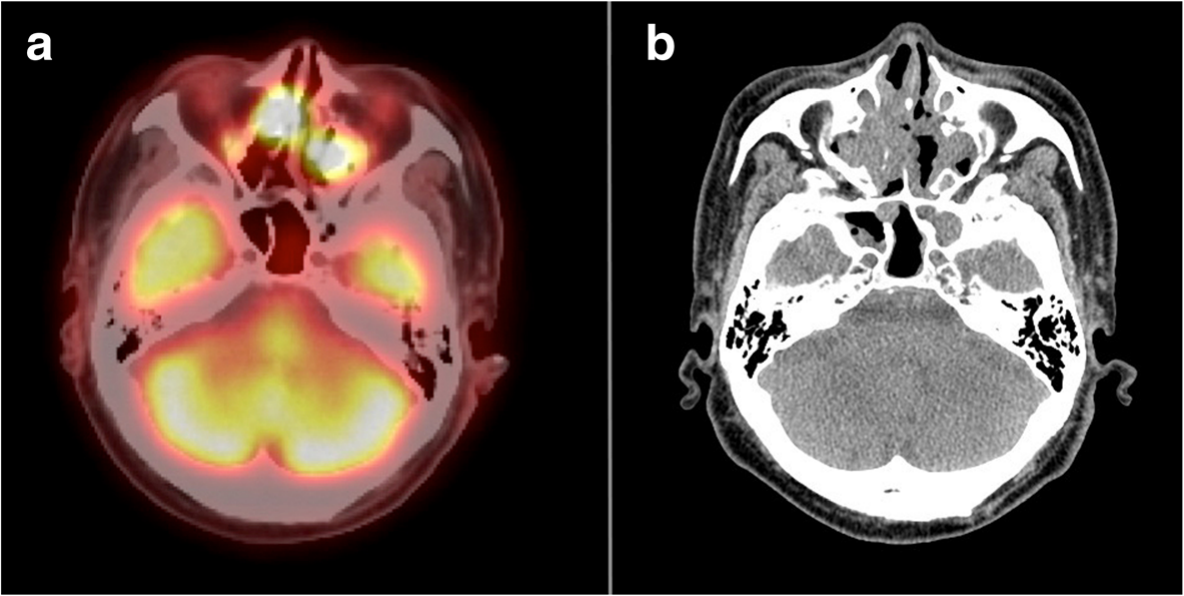
**a** Positron emission tomography/computed tomography showed abnormal uptake of ^18^F-Fluorodeoxyglucose in bilateral ethmoid sinuses. **b** Computed tomography scan showed masses in the nasal cavities and the paranasal sinuses

A 72-year-old man visited a hospital with submental tumors without B symptoms. He and his family had no history of hematologic disease. Laboratory tests showed normal blood cell counts. There were no atypical cells in the peripheral blood. Serum lactate dehydrogenase (LDH) levels were within the normal range however, the soluble interleukin-2 receptor (sIL-2R) levels were elevated (1095 U/mL). The computed tomography (CT) scan showed tumorous masses in the nasal cavities (40 × 26 mm) and the paranasal sinuses, submental masses (right 23 × 15 mm, left 19 × 11 mm), and enlarged multiple jugular lymph nodes. Positron emission tomography/CT (PET/CT) showed abnormal uptake of ^18^F-Fluorodeoxyglucose (FDG) in each lesion. The maximum standardized uptake values for the bilateral ethmoid sinuses and right submental masses were 13.0 and 4.4, respectively (Fig. 1).

**Fig. 2:**
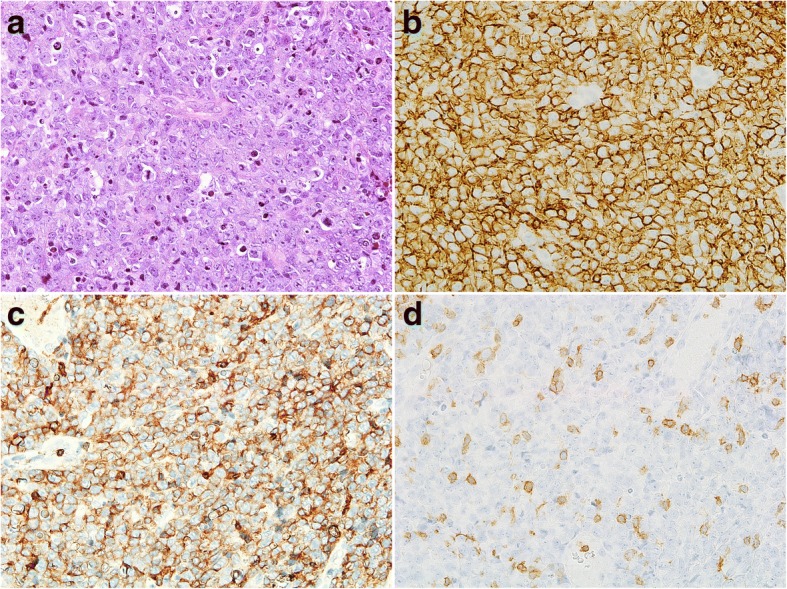
**a** Histology of the tumor in the nasal cavity. The neoplastic cells have large and round nuclei (hematoxylin and eosin staining, original magnification, × 400). The malignant cells mostly had a immunoblastic or centoblastic appearance and expressed CD20 (**b**) and CD5 (**c**) but not CD3 (**d**) (× 400)

The histology of the biopsy from nasal cavity masses showed diffuse infiltration of large lymphoid cells with centroblast-like or immunoblast-like features. In immunohistochemistry the large lymphoid cells were positive for CD20, CD79a, CD5, bcl-2, bcl-6, and MUM-1 and negative for CD3, CD10, cyclinD1, CD56, SOX11, and TIA-1 (Fig. 2). The Ki-67 labeling index was approximately 90%. Moderate level of c-myc protein was observed in about 60% of tumor cells. Weak to intermediate expression of cyclin D2 was observed in only 10% of tumor cells. In situ hybridization investigations for Epstein-Barr virus (EBV) encoded small RNA did not detect EBV. *IGH-BCL2* translocation was not detected by polymerase chain reaction. No break of *MYC* and *BCL6* were detected by fluorescent in situ hybridization. G-banding investigation showed the following karyotype: 46, XY, − 6,add(9)(p22), add(12)(p13), − 19, add(22)(q13), +mar1, and + mar2. G-banding, flow cytometry analysis, and cytological examinations on bone marrow smear specimens did not reveal involvement of lymphoma cells. Thus, a diagnosis of CD5-positive DLBCL (non-germinal center B-cell like type according to Hans algorithm) of the nasal cavity was established. After administration of 8 cycles of R-THPCOP (rituximab, pirarubicin, cyclophosphamide, vincristine and prednisolone) with 3 cycles of intrathecal chemotherapy, consisting of methotrexate and cytarabine, complete remission was achieved. Eight months after the first chemotherapy administration, local recurrences occurred in the left nasal cavity, left submental node, bilateral internal jugular nodes, and epipharynx. The patient was therefore given 4 cycles of the DeVIC regimen (dexamethasone, etoposide, ifosfamide and carboplatin) as salvage therapy.

**Fig. 3:**
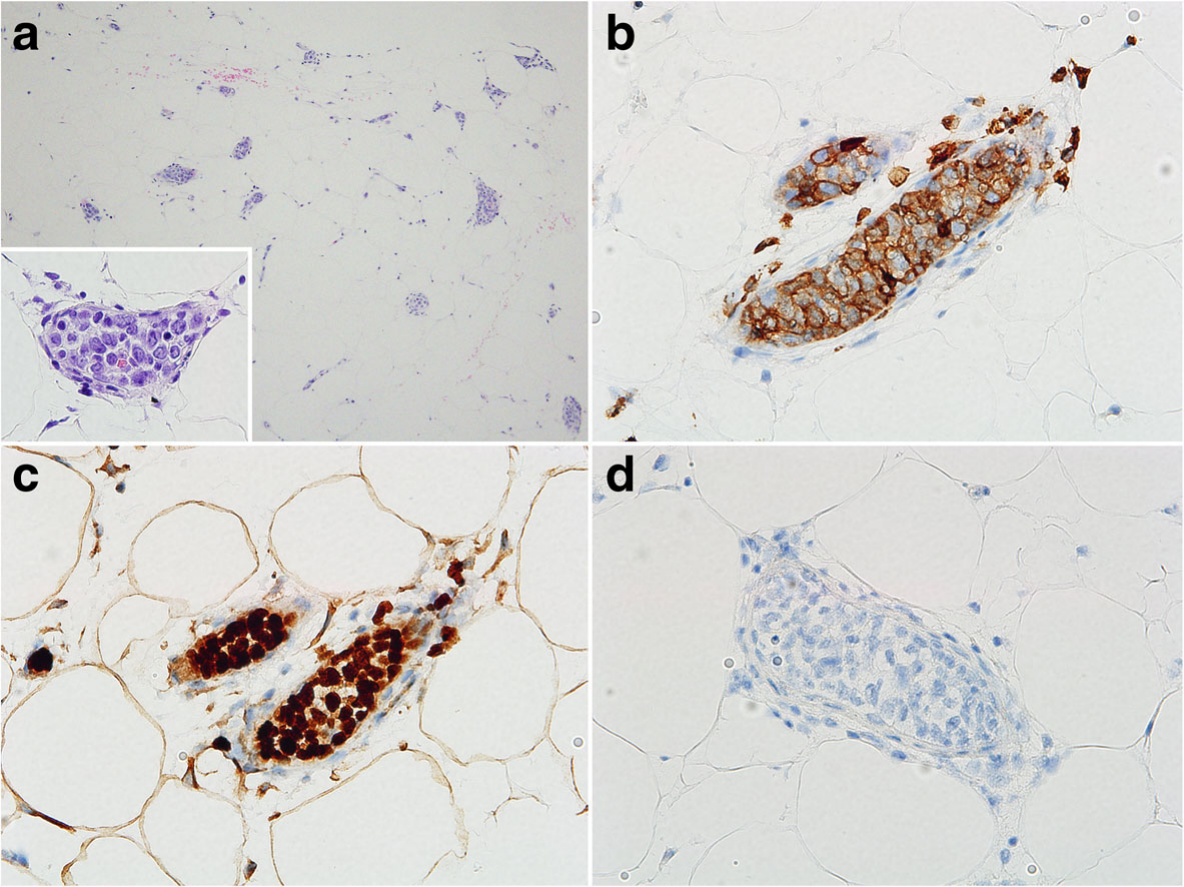
**a** Histology of random skin biopsy showing atypical cells within the lumens of the small blood vessels (hematoxylin and eosin staining, × 100 × 400). The malignant cells were morphologically similar to the cells of the primary tumor and expressed CD5 (**b**) and PAX5 (**c**) but not CD20 (**d**) (× 400)

**Fig. 4:**
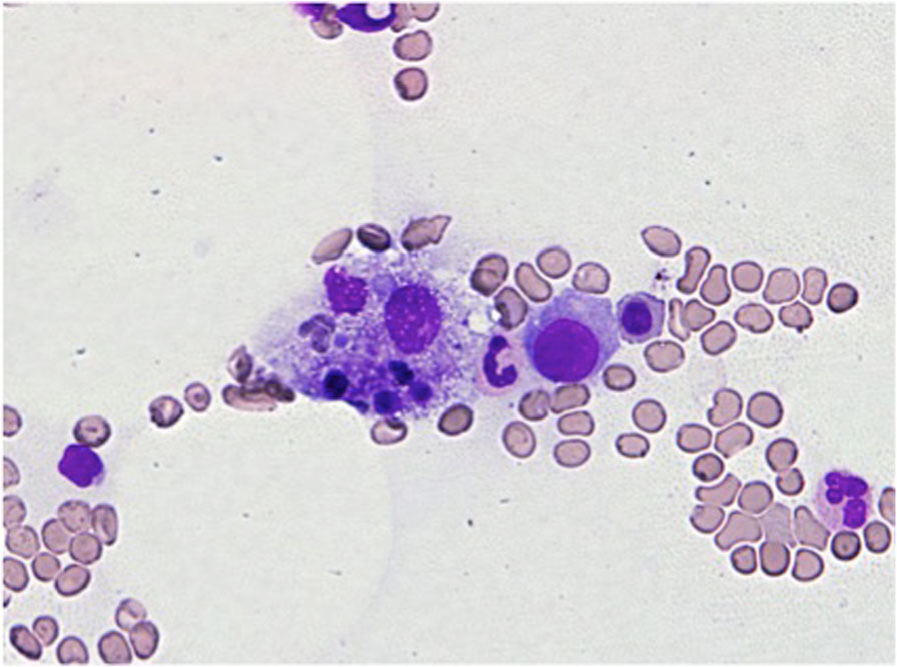
Bone marrow smears showed hemophagocytosis (Giemsa staining, × 400)

Although abnormal ^18^F-FDG uptake on PET/CT disappeared from the nasal cavities and the paranasal sinuses after the second round of therapy, the patient suffered from respiratory disturbance, fever and general fatigue without lymph node swelling. Laboratory tests showed normal white blood cell counts, anemia (hemoglobin 8.2 g/dL), and thrombocytopenia (51 × 10^3^/μL). Serum levels of LDH, sIL-2R, and ferritin were elevated (757 U/L, 5770 U/ml, and 1681 μg/L, respectively). CT images showed splenomegaly, but no tumorous lesion anywhere in the body. No apparent recurrence was observed in imaging studies, but there were elevated levels of serum markers and respiratory disturbance of uncertain cause, which clinically suggested intravascular recurrence of CD5-positive DLBCL. Therefore, a random skin biopsy was performed. The random skin biopsy of the left thigh revealed atypical large lymphoid cells within the lumens of the small blood vessels in the deep dermis and subcutis. Tumor cells were localized in intravascular spaces and did not invade the extravascular stroma. In immunohistochemical analyses, these cells showed positive expression for CD79a, PAX5, CD5, bcl-6, and MUM-1 and negative expression for CD20, CD3, CD4, CD8, and CD10 (Fig. 3). The positivity rate of cyclin D2 was less than 1%. Bone marrow smear specimens showed hemophagocytosis (HPC) (Fig. 4). According to proposed hemophagocytic lymphohistiocytosis diagnostic criteria by Filipovich et al [11], hemophagocytic lymphohistiocytosis was clinicopathologically diagnosed. Lymphoma cells were not detected in smear and histological specimens from the bone marrow biopsy, but G-banding of bone marrow cells showed complex karyotypes, suggesting a minimal residue of neoplastic cells in the bone marrow. The histological diagnosis was the Asian variant of intravascular lymphoma, which was suspected to be the purely intravascular recurrence of CD5-positive DLBCL primarily arising from nasal cavities. The patient died 3 weeks after the recurrence was diagnosed.

## Discussion and conclusions

IVL is a rare subtype of large B-cell lymphoma characterized by the exclusive or predominant proliferation of neoplastic cells within the vascular lumen [12]. A large epidemiological study showed that the age-adjusted incidence rate of IVL was 0.095 per 1,000,000 in the United States [13]. This disease is regarded as a subtype of large B-cell lymphoma according to the revised World Health Organization (WHO) classification in 2016. IVL is rapidly progressive and is often disseminated to various organs showing intravascular infiltration pattern. IVL can involve any organ, but it most commonly involves CNS, skin, lung, liver, kidneys, and adrenal glands [10, 14]. The clinical manifestations of IVL are extremely variable, such as neurological abnormalities, skin rashes, eruptions, respiratory disturbances, hepatosplenomegaly, and anemia. Interestingly, clinical manifestations differ based on geographical region and racial grouping. IVLs that occur in Western countries more commonly involve the CNS and skin, while those in Asian countries preferentially show fever, jaundice, hemophagocytic lymphohistiocytosis, hepatosplenic and bone marrow involvement, anemia, and thrombocytopenia; such cases are designated as Asian variants (Table 1) [15]. The incidence of HPC is 61% in Japanese cases of IVL [8], whereas IVL with HPC is rarely observed in Western countries (1.5% of all Western IVL cases) [15]. Ferreri et al. compared the clinical features of IVLs that were diagnosed in three different geographical areas—Western countries, Japan, and other Asian countries—by reviewing the English literature on IVL. Their study showed that IVL with HPC, which occurs in any geographical area, has similar clinical characteristics as the Asian variant described above. In addition, IVL without HPC, which occurs even in Japan, shows similar features to IVL without HPC in Western and other Asian countries. Therefore, the authors concluded that differences in the clinical features of IVL seem to be correlated with concomitant presence of HPC, rather than the geographical area where the lymphoma is diagnosed [15]. Despite the differences in clinical presentation between the two groups of IVL there are no significant differences in terms of patient survival [8, 16].

**Table 1:** Comparison of clinical features and laboratory findings between Western IVL and Asian variant IVL

	Incidence	
	Western IVL	Asian variant IVL
Clinical features		
Fever	Low	High
Jaundice	Very low	Low
Hepatosplenic involovement	Low	High
Bone marrow involvement	Low	High
Cutaneous lesion	Low	Very low
Neurological involvement	Low^a^	Low^a^
Laboratory findings		
Anemia	High^b^	High^b^
Thrombocytopenia	Low	High
High serum LDH levels	High^b^	High^b^
High ALT levels	Very low	Low
High bilirubin levels	Very low	Low

*IVL* Intravascular lymphoma, *LDH* Lactate dehydrogenase, *ALT* Alanine aminotransferase (modified from Ferreri et al. [15])

Very low: Less than 10%, Low: 10–50%, High: more than 50%

^a^More common in Western IVL

^b^More common in Asian variant IVL

CD5-positive DLBCL is recognized as a subset of DLBCL. Some cases of CD5-positive DLBCL are not preceded by any other lymphoproliferative diseases (de novo CD5-positive DLBCL), though most cases secondarily arise in CLL (Richter syndrome) [1]. De novo CD5-positive DLBCL constitutes 5–22% of all DLBCL cases [1, 2, 5]. Although de novo CD5-positive DLBCL is not included as a distinct subtype of large B-cell lymphoma in the revised WHO classification of 2016, it has been reported to have unique clinicopathologic features and very poor outcomes with frequent relapse [2, 4, 5, 7]. Western groups have reported that CD5-positive DLBCL patients have a significantly worse overall survival rate than CD5-negative DLBCL patients (5-year overall survival rates of 35.5 and 64.8%, respectively) [2]. Although in our case cyclin D2 was almost negative in both the primary and recurrent lesions, a previous study showed that cyclin D2 was overexpressed more frequently in de novo CD5-positive DLBCL than in CD5-negative DLBCL (98 and 28%, respectively) [17]. In addition, several studies using gene expression profiling and immunohistochemical stainimg have shown cyclin D2 (*CCND2*) to be an independent indicator of an inferior survival in DLBCLs [18, 19], which suggests that the biological aggressiveness and worse prognosis of CD5-positive DLBCL may be attributed to overexpression of cyclin D2. The most common site of extranodal involvement of de novo CD5-positive DLBCL is the bone marrow and it frequently presents with intravascular or intrasinusoidal infiltration patterns [5], which are similar to the clinicopathologic features of the Asian IVL variant. Interestingly, the frequency of CD5 expression is higher in the Asian IVL variant than in the common type of DLBCL. Thus, CD5-positive DLBCL shares common clinicopathologic features with IVL, suggesting a close relationship between these two diseases.

In our case, CD5-positive DLBCL primarily developed in nasal cavities; first and second chemotherapy might have aided in eliminating the majority of tumor cells. Few residual lymphoma cells, especially the cells that preferentially proliferate while anchoring to the vascular lumen, might expand and be transferred to vascular wall floors in the lungs and skin, resulting in IVL. A similar case with cutaneous CD5-positive DLBCL that disappeared spontaneously and relapsed 2 years later as CD5-positive IVL was described in one Japanese report. This previous case and our case suggest that IVL might be a representative manifestation of advanced-stage of CD5-positive DLBCL. The recurrent tumors in our case demonstrated only an intravascular component in histology but did not display any tumorous lesions or lymph nodal swelling in imaging investigations. There have been some reported cases of IVL complicated with other types of lymphoma or CLL, but the number of reported cases who developed pure IVL following prior B-cell lymphoma are limited [20–23].

We have reported an interesting case that primarily arose as CD5-positive DLBCL in the nasal cavities and relapsed as pure IVL with hemophagocytic lymphohistiocytosis. Our case suggests that de novo CD5-positive DLBCL is closely related to IVL and that the clinicopathological features of these two diseases might overlap considerably. To explore the pathogenesis of CD5-positive DLBCL and IVL and to establish a more effective therapeutic strategy for these diseases, genetic and epigenetic profiling of CD5-positive DLBCL and IVL must be examined.

## Abbreviations

CLL: Chronic lymphocytic leukemia
CNS: Central nervous system
CT: Computed tomography
DLBCL: Diffuse large B-cell lymphoma
EBV: Epstein-Barr virus
FDG: Fluorodeoxyglucose
HPC: Hemophagocytosis
IVL: Intravascular large B-cell lymphoma
LDH: Lactate dehydrogenase
PET/CT: Positron emission tomography/computed tomography
sIL-2R: Soluble interleukin-2 receptor
WHO: World Health Organization

## References

[1] Matolcsy A, Chadburn A, Knowles DM (1995). De novo CD5-positive and Richter's syndrome-associated diffuse large B cell lymphomas are genotypically distinct. Am J Pathol.

[2] Xu-Monette ZY, Tu M, Jabbar KJ, Cao X, Tzankov A, Visco C (2015). Clinical and biological significance of de novo CD5+ diffuse large B-cell lymphoma in western countries. Oncotarget.

[3] Miyazaki K, Yamaguchi M, Suzuki R, Kobayashi Y, Maeshima AM, Niitsu N (2011). CD5-positive diffuse large B-cell lymphoma: a retrospective study in 337 patients treated by chemotherapy with or without rituximab. Ann Oncol.

[4] Yamaguchi M, Nakamura N, Suzuki R, Kagami Y, Okamoto M, Ichinohasama R (2008). De novo CD5+ diffuse large B-cell lymphoma: results of a detailed clinicopathological review in 120 patients. Haematologica.

[5] Yamaguchi M, Seto M, Okamoto M, Ichinohasama R, Nakamura N, Yoshino T (2002). De novo CD5+ diffuse large B-cell lymphoma: a clinicopathologic study of 109 patients. Blood.

[6] Tagawa H, Suguro M, Tsuzuki S, Matsuo K, Karnan S, Ohshima K (2005). Comparison of genome profiles for identification of distinct subgroups of diffuse large B-celllymphoma. Blood.

[7] Niitsu N, Okamoto M, Tamaru JI, Yoshino T, Nakamura N, Nakamura S (2010). Clinicopathologic characteristics and treatment outcome of the addition of rituximab to chemotherapy for CD5-positive in comparison with CD5-negative diffuse large B-cell lymphoma. Ann Oncol.

[8] Murase T, Yamaguchi M, Suzuki R, Okamoto M, Sato Y, Tamaru J (2007). Intravascular large B-cell lymphoma (IVLBCL): a clinicopathologic study of 96 cases with special reference to the immunophenotypic hererogeneity of CD5. Blood.

[9] Murase T, Suzuki R, Yamaguchi M, Okamoto M, Sato Y, Tamaru J (2004). High incidence of Asian variant intravascular large B-cell lymphoma (IVL) among IVL in Japan: Clinicopathologic study of 95 patients. Blood.

[10] Yegappan S, Coupland R, Arber DA, Wang N, Miocinovic R, Tubbs RR (2001). Angiotropic lymphoma: an immunophenotypically and clinically heterogeneous lymphoma. Mod Pathol.

[11] Filipovich AH. Hemophagocytic lymphohistiocytosis (HLH) and related disorders. Hematol Am Soc Hematol Educ Program. 2009:127–31. 10.1182/asheducation-2009.1.127. 10.1182/asheducation-2009.1.127 20008190

[12] Swerdlow SH, Campo E, Harris NL, Jaffe ES, Pileri SA, Stein H (2017). WHO classification of Tumours of Haematopoietic and lymphoid tissues.

[13] Rajyaguru DJ, Bhaskar C, Borgert AJ, Smith A, Parsons B (2017). Intravascular large B-cell lymphoma in the United States (US): a population-based study using surveillance, epidemiology, and end results program and National Cancer Database. Leuk Lymphoma..

[14] Ferreri AJ, Campo E, Seymour JF, Willemze R, Ilariucci F, Ambrosetti A (2004). Intravascular lymphoma: clinical presentation, natural history, management and prognostic factors in a series of 38 cases, with special emphasis on the 'cutaneous variant. Br J Haematol.

[15] Ferreri AJ, Dognini GP, Campo E, Willemze R, Seymour JF, Bairey O (2007). Variations in clinical presentation, frequency of hemophagocytosis and clinical behavior of intravascular lymphoma diagnosed in different geographical regions. Haematologica.

[16] Ponzoni M, Ferreri AJ, Campo E, Facchetti F, Mazzucchelli L, Yoshino T (2007). Definition, diagnosis, and management of intravascular large B-cell lymphoma: proposals and perspectives from an international consensus meeting. J Clin Oncol.

[17] Igawa T, Sato Y, Takata K, Iwaki N, Tanaka T, Asano N (2013). De novo CD5-positive diffuse large B-cell lymphomas show high specificity for cyclin D2 expression. Diagn Pathol.

[18] Lossos IS, Czerwinski DK, Alizadeh AA, Wechser MA, Tibshirani R, Botstein D (2004). Prediction of survival in diffuse large-B-cell lymphoma based on the expression of six genes. N Engl J Med.

[19] Hans CP, Weisenburger DD, Greiner TC, Chan WC, Aoun P, Cochran GT (2005). Expression of PKC-beta or cyclin D2 predicts for inferior survival in diffuse large B-cell lymphoma. Mod Pathol.

[20] McKelvie PA, Wools C, Roberts L, Cook M (2013). Intravascular large B-cell lymphoma. occurring 25 years after treatment of ALK-positive anaplastic large cell lymphoma. Leuk Lymphoma.

[21] Puckrin R, Pop P, Ghorab Z, Keith J, Chodirker L, Lin Y (2017). Intravascular large B-cell lymphoma presenting as Richter's syndrome with cerebral involvement in a patient with chronic lymphocytic leukemia. Clin Case Rep.

[22] Katz DA, Miller IJ, Gregory SA (2010). Intravascular B-cell lymphoma following nodal diffuse large B-cell lymphoma. Clin Adv Hematol Oncol.

[23] Kasuya A, Hashizume H, Takigawa M (2011). Early diagnosis of recurrent diffuse large. B-cell lymphoma showing intravascular lymphoma by random skin biopsy. J Dermatol.

